# A Single Center Experience with the Use of Occlunix Septal Occluder for Closure of Atrial Septal Defects^☆^

**DOI:** 10.1016/j.ihj.2026.04.014

**Published:** 2026-04-22

**Authors:** N. Sharath, Anjana S. Jayan, Kavassery Mahadevan Krishnamoorthy, Deepa Sasikumar, Arun Gopalakrishnan

**Affiliations:** Department of Cardiology, Sree Chitra Tirunal Institute for Medical Sciences and Technology (SCTIMST), Thiruvananthapuram, Kerala, India

**Keywords:** Atrial septal defect, Congenital heart disease, Device closure, Transcatheter intervention, Occlunix

## Abstract

The Occlunix® septal occluder (OSO) is a new generation double disk occluder device for catheter closure of the secundum atrial septal defect (ASD). Fifty-six consecutive patients who underwent ASD device closure with the OSO between February 2022 and April 2024 were evaluated in this single-center observational study. All procedures were done under transesophageal echocardiography guidance. The OSO was successfully deployed in all patients (technical success 100%). Fifty-five patients had complete closure of the intervened ASD after device closure (procedural success 98.2%). The sole patient with trivial intradevice shunt had complete seal of the defect at 1-month follow-up. There were no device related complications – cardiac, vascular or systemic. During the follow-up period of 32.2 ± 9.1 months, the device position was satisfactory in all, with no complications. The OSO was associated with lower procedural cost than one of the most used conventional ASD occluders used in the country.

## 1 Introduction

Transcatheter device closure is the established standard of care for secundum atrial septal defects (ASDs) in indicated patients whenever the anatomy is deemed feasible by echocardiographic imaging. The Occlunix® septal occluder (OSO) is a novel ASD device developed by Rivarp Medical Pvt, Ltd, an Indian company based in Bengaluru, Karnataka. This device has been approved by the Central Drugs Standard Control Organization (CDSCO). The device has a similar design and construction to Amplatzer septal occluder (ASO) (Abbott Laboratories, Chicago, IL, USA), with minor improvements aimed at increasing the safety of catheter ASD closure.

## 2 Methods

This is a single center retrospective study that evaluated the safety and efficacy of transcatheter ASD device closure using the OSO. All consecutive patients diagnosed with secundum ASD and volume overload of the right heart chambers and were considered for device closure with the OSO between February 2022 and April 2024 were included in this study. The choice of ASD occluder depended on the discretion of the primary operator. The study was approved by the Institute Ethics Committee. Written informed consent was taken from the patients and parent/immediate relative for the procedure. Statistical analysis was performed on IBM SPSS statistics (version 30).

The OSO is a self-expandable, double-disc device made from nitinol wire mesh. The frame of the disc provides a foundation for the growth of tissues. Three polyester fabric inserts help in immediate occlusion (Fig. 1). The non-allergic, polyester fabric promotes total occlusion of the defect. Waist diameters range from 6 to 50 mm with 23 different sizes available. The device and cable are marketed separately. The OSO is screwed onto a 6F delivery cable and slenderized into a short loader. The device is deployed via a 45° Occlunix® delivery system ranging from 7 to 14F.

**Fig. 1:**
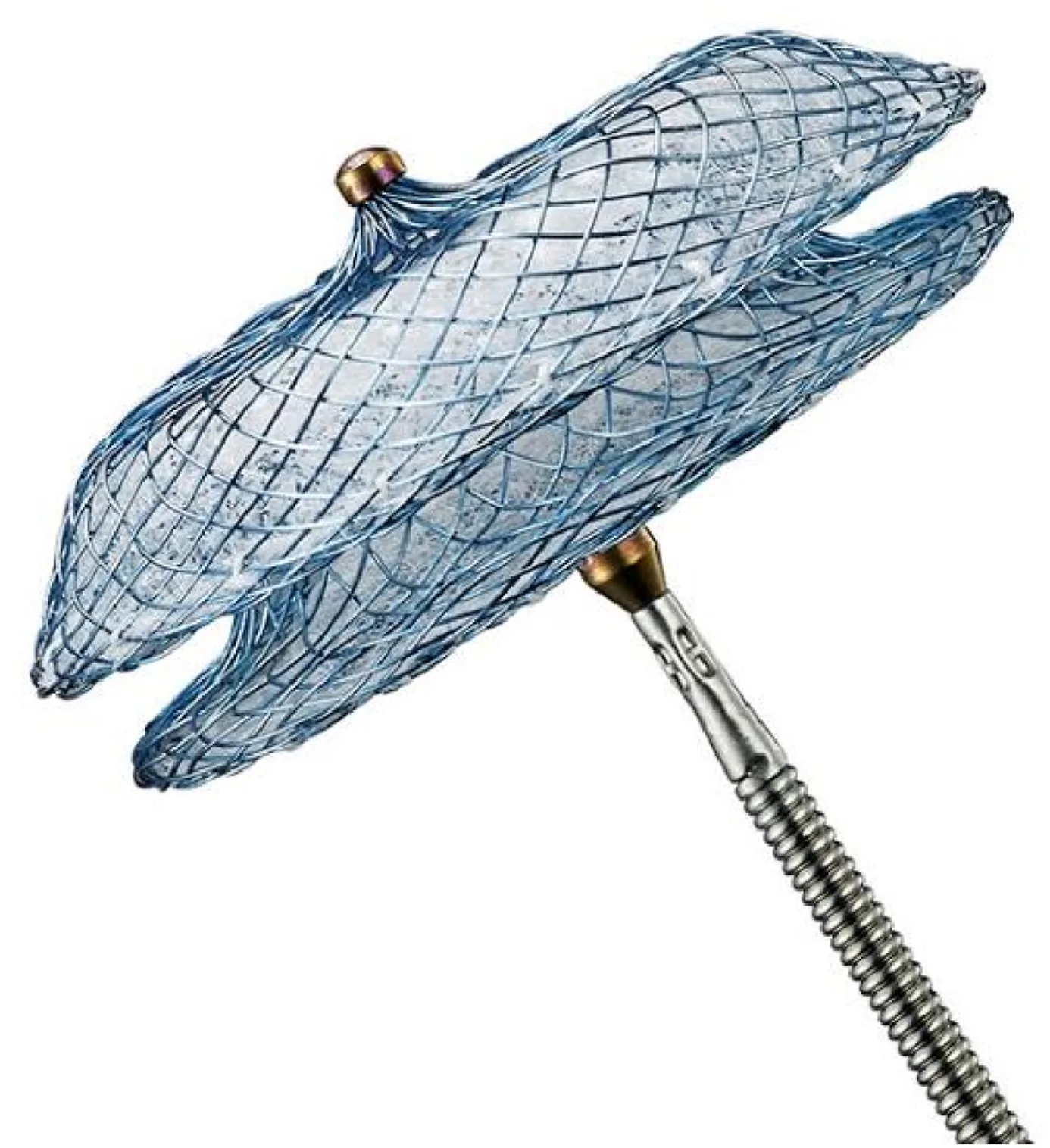
Side view of the OSO.

## 3 Results

Fifty-six patients who were taken for transcatheter ASD device closure with the OSO constituted the study cohort (Table 1). The median age was 26 years. The mean ASD diameter was 18.8 ± 6.13 mm, and the occluder used measured 21.1 ± 5.9 mm.

**Table 1:** Baseline characteristics.

Parameter	Statistic^a^
**Age (years), Median (IQR)**	26 (10.25 – 44.75)
**5**–**10 years, n (%)**	14 (25%)
**11**–**18 years, n (%)**	7 (12.5%)
**18**–**60 years, n (%)**	33 (58.9%)
**>60 years, n (%)**	2 (3.5%)
**Female gender, n (%)**	43 (76.7%)
**Height (cm), Median (IQR)**	153 (125 – 160)
**Weight (kg), Median (IQR)**	49 (23 – 61.25)
**Pulmonary hypertension, n (%)**	22 (39.2%)
**Estimated pulmonary artery systolic pressure^#^, Median (IQR)**	27.5 (10 – 60)
**Diabetes mellitus, n (%)**	3 (5.3%)
**Systemic hypertension, n (%)**	2 (3.5%)
**Atrial fibrillation, n (%)**	0
**PR interval, Median (IQR)**	160 (140 – 170)
**Prolonged PR interval, n (%)**	4 (7.1%)
**QRS axis, Median (IQR)**	85 (60 – 90)
**ASD maximum diameter (mm) Mean ± SD**	18.8 ± 6.13
**Device diameter (mm) Mean ± SD**	21.1 ± 5.9
**Eccentricity index^#^**Median(IQR)	0.51 (0.08 – 0.66)
**Delivery system size (F) Median (IQR)**	10 (9 – 11)
**Guidance**	
**TEE only, n (%)**	22 (39.2%)
**TEE + Fluoroscopy, n (%)**	34 (60.7%)
**RUPV approach, n (%)**	8 (14.2%)
**LA roof technique, n (%)**	3 (5.4%)
**Immediate complete occlusion, n (%)**	55 (98.2%)
**Complications**	0
**Aspirin, n (%)**	56 (100%)
**Oral anticoagulation**	0

^#^Pulmonary artery systolic pressure derived by analysis of tricuspid regurgitation on transthoracic echocardiography (4 x peak tricuspid regurgitation velocity) + estimated right atrial mean pressure by analysis of inferior vena cava.

#Eccentricity index calculated using the parameters of an ellipse.^3^

a Mean ± SD used for normal data, Median (IQR) displayed for data not conforming to normal distribution.

The OSO was successfully implanted in all 56 patients (technical success 100%). 39.2% of procedures were totally done under transesophageal echocardiography (TEE) guidance with zero fluoroscopy from the conventional approach. Patients with any degree of pulmonary hypertension underwent right heart catheterization and constituted the main indication for a fluoroscopy-guided procedure. No patient had severe pulmonary hypertension. Support techniques were used for difficult anatomy and done under fluoroscopy guidance. The device was successfully deployed on the first attempt in 49 patients (87.5%). Prolapse of the left atrial disc into the right atrium was the reason for the multiple attempts in 7 patients.

Fifty-five patients had complete closure of the intervened ASD documented on intraprocedural TEE after device closure (procedural success 98.2%). One patient (1.8%) had a trivial residual shunt immediately following the procedure, which sealed off completely at 1-month follow-up. Another patient was diagnosed with an additional 3 mm ASD on pre-procedural TEE which was kept for medical follow-up while the primary defect was successfully closed by device. All patients received Aspirin for six months post-procedure.

All procedures were uneventful with no device-related complications including embolization, conduction disturbances, tachy- or brady arrhythmia, vascular complications, injury to any cardiac chamber, pericardial effusion, bleeding or thrombotic complications, fever or allergic manifestations. There was no instance of device malformation during or after the procedure.

There was no residual shunt across the intervened ASD in any patient at 1 month, 6 month, and 1-year follow up. During a follow-up of 32.2 ± 9.1 months, all devices were documented to be in their appropriate positions with no interference to adjacent cardiac structures. There was no evidence of thrombus formation, endocarditis, or hemolysis. No patient developed device-related erosion, valve regurgitation, allergic reactions to nickel, or atrioventricular conduction disturbances. No neurological events occurred. Follow-up was complete in all patients, and all patients remained asymptomatic at their latest follow-up.

The OSO and its delivery system are marketed at a price 28% lower than the ASO, one of the most used ASD devices in the country. The final hardware bill to the patient at our institute was 3.2% lower with the OSO as opposed to the ASO [₹72,058 (70,077–77,269) vs ₹75,413 (71,364–80,326) p = 0.001]. There was no difference in the costs pertaining to other heads including intensive care charges, ward charges, or miscellaneous expenses. The duration of total hospital stays, and post-procedural stay in the intensive care with the OSO was not different from that of the other device.

## 4 Discussion

The self-expandable, double-disk design of the OSO is an improvisation on conventional ASD occluders. While the dimensions of the right and left atrial discs are like the ASO, the OSO waist lengths of 26 mm and larger occluders are marginally longer than the ASO.

The relatively larger waist length of the larger OSO devices is a potential seating advantage in percutaneous closure of large malaligned defects. This is likely reflected in the good results observed in a recent report of the device-assisted device closure of ASD sized 42 mm and larger with the OSO.^1^ Septal malalignment constitutes a risk factor for erosion and device embolization primarily because of the impingement of the device disc into the aortic wall when the occluder wedges itself between the septum primum and the septum secundum.^2^

The cost-effectiveness of the OSO has noteworthy implications for low-middle income countries where the out-of-pocket expenditure is a major concern in optimal delivery of health care services with its implications in cardiovascular outcomes.

The small sample size and a single-center retrospective study design are major limitations of our study. However, there was no attrition in our study. The choice of occluder depended on the treating cardiologist, and the possibility of bias cannot be ruled out.

## 5 Conclusions

This single-center observational study suggests excellent technical success, procedural and clinical outcomes with the OSO over a 2.5-year follow up period. The OSO is associated with lower procedure costs than the ASO. Larger multicentric prospective studies are needed to validate our findings for generalizability.

## Patient consent for publication

Obtained.

## Ethical considerations

The study was conducted in accordance with the ethical standards laid down in the 1964 Declaration of Helsinki and its amendments. Informed written consent was taken from the patients concerned. No patient identity particulars have been disclosed.

## Data availability statement

All data pertaining to this study are presented along with the manuscript.

## Ethical approval statement

Obtained from the Institutional Ethics Committee (IEC number – SCT/IEC/2321/NOVEMBER-2024).

## Declaration

This manuscript did not involve the use of generative AI or AI-assisted technologies in the preparation or alteration of the manuscript, tables, or images.

## Funding

This was an investigator-initiated non-funded study.

## Declaration of competing interest

The authors declare that they have no known competing financial interests or personal relationships that could have appeared to influence the work reported in this paper.
